# miRToolsGallery: a tag-based and rankable microRNA bioinformatics resources database portal

**DOI:** 10.1093/database/bay004

**Published:** 2018-02-19

**Authors:** Liang Chen, Liisa Heikkinen, ChangLiang Wang, Yang Yang, K Emily Knott, Garry Wong

**Affiliations:** 1 Faculty of Health Sciences, University of Macau, E12 Avenida da Universidade, Taipa, Macau S.A.R., China; 2 Department of Biological and Environmental Science, University of Jyväskylä, FI-40014, Jyväskylä, Finland

## Abstract

Hundreds of bioinformatics tools have been developed for MicroRNA (miRNA) investigations including those used for identification, target prediction, structure and expression profile analysis. However, finding the correct tool for a specific application requires the tedious and laborious process of locating, downloading, testing and validating the appropriate tool from a group of nearly a thousand. In order to facilitate this process, we developed a novel database portal named miRToolsGallery. We constructed the portal by manually curating > 950 miRNA analysis tools and resources. In the portal, a query to locate the appropriate tool is expedited by being searchable, filterable and rankable. The ranking feature is vital to quickly identify and prioritize the more useful from the obscure tools. Tools are ranked via different criteria including the PageRank algorithm, date of publication, number of citations, average of votes and number of publications. miRToolsGallery provides links and data for the comprehensive collection of currently available miRNA tools with a ranking function which can be adjusted using different criteria according to specific requirements.

**Database URL**: http://www.mirtoolsgallery.org

## Introduction

MicroRNAs (miRNAs) represent a class of 21–22 nucleotide long, small non-coding RNAs. These miRNA transcripts are derived from the nucleus as primary transcripts (pri-miRNAs) which are then cleaved to shorter precursor miRNAs (pre-miRNAs). Pre-miRNAs are exported into cytoplasm via exportin and after RNA endonuclease Dicer activity, processed to become mature miRNAs. Functionally, miRNAs are loaded into the RNA silencing complex to degrade target mRNA (1). MiRNAs are found in animals, plants and viruses and act as important biological regulators in a wide range of physiologic processes such as cell proliferation, differentiation, apoptosis and embryonic development (1). Moreover, miRNAs contribute towards multiple diseases (2, 3).

Many bioinformatics tools have been developed to study miRNAs, including tools for miRNA discovery, miRNA target prediction, miRNA regulatory network identification and for combining miRNA target prediction with miRNA and mRNA expression data (4). These tools cover databases, web services and stand-alone software. Since the early miRNA related tools were limited to a single purpose, integrated analysis tools and meta-server tools are now emerging. Due to the large number and multifunction of these miRNA tools, finding a suitable one for a particular research question is challenging. Some databases have been built to collect miRNA tools in order to help researchers to quickly find the appropriate one. Non-coding RNA Databases Resource (NRDR) is a guided web resource for non-coding RNA databases and includes 70 miRNA related tools (5). Tools4miRs currently gathers about 198 methods for broadly defined miRNA analysis (6). miRandb is an online database of 188 miRNA-related bioinformatics tools (7). Overall, it is difficult to navigate across those miRNA analysis resources due to the vast number and some insufficiently specific databases. Moreover, the user may have no way of knowing how useful these resources may be, except by trial and error. In the present work, we built a portal linking to a powerful database with tagged and rankable features containing a comprehensive set of miRNA data analysis tools.

miRToolsGallery aims to make it easier for researchers to find the appropriate miRNA tools for their research. To our knowledge, miRToolsGallery is, by far, the most comprehensive collection of miRNA tools available and it allows users from diverse backgrounds to rank tools, to filter tools by taxonomy terms and finally, to aid them to select optimal tools for their projects according to their specified criteria.

## Materials and methods

### Data collection, inclusion and exclusion criteria

**Figure 1.**
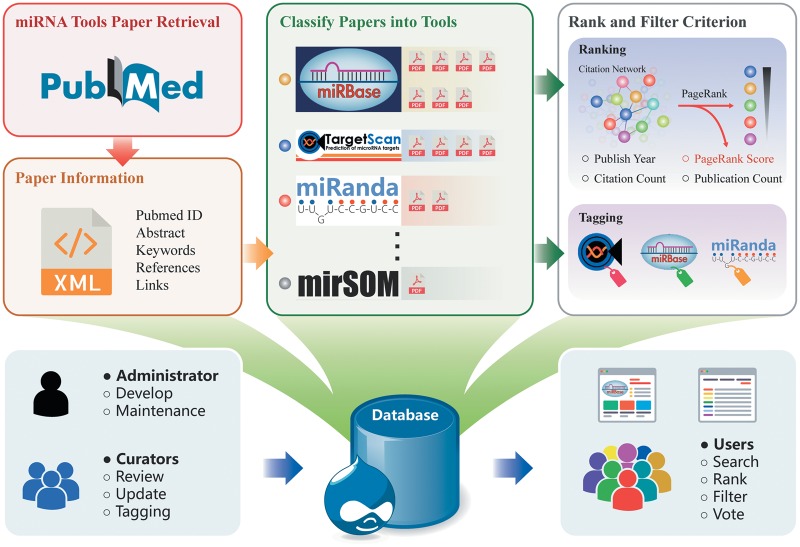
Overview of miRToolsGallery architecture. First, miRNA tools are manually curated from PubMed and basic information is extracted. Second, papers are catalogued based on each tool. Finally, calculations of rank criterion and tagging the tools are performed in the back end. In miRToolsGallery, the administrator is in charge of development and maintenance of the database. Curators collect, upload and label the tools in the system. Anonymous users are end users that can freely filter, rank and search the tools. Their improvement suggestions or reports provide the updates and basis for future versions of miRToolsGallery.

Figure 1 shows the workflow of building the database including data collection, tools tagging, tools ranking and use cases. We reviewed the literature published up to September 2017 to search for relevant publications on miRNA tools through PubMed (http://www.ncbi.nlm.nih.gov/pubmed/). We used the following search command, where Boolean operators (AND, OR, NOT) combine the keywords: (((((miRNA) OR microRNA) OR ‘small RNA’) OR ‘small non-coding’)) AND ((((((((((database) OR webserver) OR http) OR website) OR ‘source code’) OR https) OR www) OR pipeline) OR workflow) OR script), which gave about 3000 results. Additionally, we collected tools from review papers (4, 8, 9), and bioinformatics code collection and distribution centres: CRAN (https://cran.r-project.org/), Bioconductor (10) and GitHub (https://github.com/) using miRNA or microRNA as the search term. miRToolsGallery aims to cover all bioinformatics tools for miRNA analysis, from large and complex platform/pipelines such as Chipster (11) and UEA small RNA workbench (12), to a simple specific function Python script, such as a miRNA functional enrichment analysis script (13). However, some authoritative comprehensive databases such as NCBI (https://www.ncbi.nlm.nih.gov/), Ensemble (14) and KEGG (15) were not included. In addition, we excluded all published algorithms and pipelines that did not have any directly usable resources online. Finally, we manually curated 1170 articles describing 970 miRNA tools in the first and current version of miRToolsGallery. For citation network data, we extracted the information from PubMed by CRAN R package rentrez (https://cran.r-project.org/web/packages/rentrez/index.html). The last query was performed on 10 September 2017, from PubMed bibliographic record and the DbBuild code was Build170910-2332 m.6. See red and orange frame in Figure 1.

The descriptions of tag terms were linked to Wikipedia (https://en.wikipedia.org) or their official websites and the introduction for each tool was directly obtained from the PubMed publication abstract if available. External curators can also manually curate the information of the tools and check whether resource links are active, and submit information to miRToolsGallery. The current version only contains tools curated by the authors. miRToolsGallery is freely available at http://www.mirtoolsgallery.org.

### Tagging miRNA tools

miRToolsGallery is a tag-based database. In the back-end layer, we implemented a tagging module to classify the tools: one tool can have many tags, and one tag can belong to many tools. Tags contribute to systematically classify miRNA tools and help the user to filter the available tools.

We defined six unique tag groups: ‘Status,’ ‘Implementation technology,’ ‘Platform,’ ‘Species,’ ‘Methods’ and ‘Tags.’ The definitions of the tags were as follows: (a) ‘Status’ marks the availability of the tools. If the resource link (web server or source code) was deprecated, it was labelled as ‘dead,’ if not, ‘active’ was used. Because many tools have moved to a new web address (different from the link in the original published paper), we conscientiously marked this label after deeply searching for the tools. (b) ‘Platform’ tells if the tool runs on a web server or needs to be downloaded to a local system. All the tools developed as a database or a web server that can be used through a browser were grouped as ‘web based,’ and the other, stand-alone programs were tagged by ‘Windows,’ ‘Linux/Unix’ or ‘Mac OS’ according to the operating system. (c) ‘Species’ tags whether the tools are restricted for use with a particular organism. Only the most popular species with a common name have their own tags and the rest are marked as ‘Other Species.’ (d) ‘Implementation Technology’ is for tagging the application development technique or programming language, such as Perl, PHP and R. (e) ‘Methods’ gives the algorithm applied in the tool, for example ‘Support Vector Machine’ or ‘Random Forest’ (16, 17). (f) The largest and comprehensive group ‘Tags’ comprise keywords extracted from the original publications, for example ‘miRNA prediction’ and ‘Integrated analysis.’ ‘Tags’ were manually curated by removing and unifying any repetitions.

### Ranking miRNA tools

The database provides five ranking options: latest publication year, PubMed citation count, publication count, average vote and PageRank score based on tools citation network.

The definitions of the ranking options were: (a) latest publication year, is the latest publication year of the tool; (b) PubMed citation count, is the total citations of all publications for a tool; (c) Publication count, equals to how many published papers the tool has; (d) Average votes, we allow every user, both register and visitor, to vote for the tools and then we will calculate the average of the total user votes in real time and (e) PageRank score, gives the tools popularity in the miRNA research community based on the tools citation network.

The PageRank algorithm was developed by Google (18), and originally used for sorting the retrieval results of the search engine. Recently, the PageRank algorithm has been used to rank the importance of research literature (19). However, the literatures citation network is an acyclic directed graph since former published papers cannot cite latter papers. So, it is not suitable for applying the PageRank algorithm (18, 20). We improved this by integrating the citation information of literature for the tools, since it is reasonable to rank the tools, but not the papers. We merged all published articles related to one tool to represent the tool node in the network. In Figure 1, green and grey frame gave an overview of merging papers into tool as a node in the network. Now the directed tools citation network is suitable to apply the PageRank algorithm.

*R* is a vector that holds the rank of each tool. $r_{i}$ is the rank of the *i*-th tool. *N* is the total number of tools in miRToolsGallery.

(1) $$R={\left(r_{1},r_{2},\ldots ,r_{N}\right)}^{T}$$

The initial rank value of each tool is set to $\frac{1}{N}$, so $R_{0}={\left(\frac{1}{N},\frac{1}{N},\ldots ,\frac{1}{N}\right)}^{T}$.

The citation matrix *C* is:

(2) $$C=[\begin{array}{ccccc} c_{11} & \cdots & c_{1j} & \cdots & c_{1n}\\ \;\vdots & & \;\vdots & & \;\vdots \\ c_{i1} & \cdots & c_{ij} & \cdots & c_{in}\\ \;\vdots & & \;\vdots & & \;\vdots \\ c_{n1} & \cdots & c_{nj} & \cdots & c_{nn} \end{array}]$$

*C* is a sparse square matrix, and $c_{ij}$ represents how many times the *i*-th tool cites the *j*-th tool. So the equation of PageRank is as follows:

(3) $$R_{i}=[\frac{\alpha }{N}\cdot I+(1-\alpha )\cdot C]\cdot R_{i-1}$$

α is a small floating number, and *I* is a unit matrix. We applied R package igraph to implement the PageRank algorithm in this study (21).

### Database construction

miRToolsGallery is literature based. We tracked each tool by its unique PubMed ID, and reserved the papers affiliated to any new version of it in the database for tracking its activity as well. In-house R and Perl scripts were used to process data for bulk upload to the database. These scripts are available in Supplementary Material.

The whole system was developed via Drupal 7 (https://www.drupal.org/), a free and open source content management system. The running environment was constructed by XAMPP on Ubuntu Linux system, an all in one package that contains Apache, MySQL and PHP. The database was hosted in the Amazon Web Services EC2 instance. The website is user-friendly and works well on PC, but also can be easily accessed via smart phone and pad with a good user experience.

## Results

### Database

**Figure 2.**
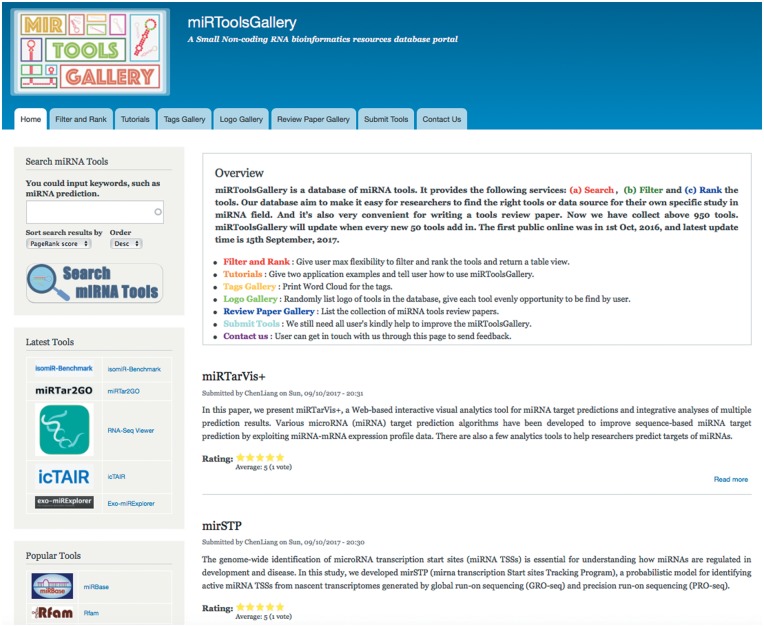
The homepage of miRToolsGallery. Bottom of banner is the navigation of the website, including Home, Filter and Rank, Tutorial, Tags Gallery, Logos Gallery, Review Paper Gallery, Submit Tools and Contact Us. The first left sidebar holds the Search Input. The second left sidebar is the tools list which by default is ranked by latest published year. The third left sidebar is the tools list which is default ranked by popularity (PageRank score). The top of content region is the brief introduction of miRToolsGallery and version information followed by the list of new submitted miRNA tools.

The organizational structure of miRToolsGallery can be seen in Figure 1. There are three roles in our system: administrator, curator and anonymous guest user. The administrator has the highest authority in this system and is in charge of developing and maintaining the system. The curators can input the basic information of the tools, review, modify and tag the tools. Biologists or bioinformaticians interested in miRNA research are our potential users. The aim is that every user can easily find the appropriate tool for their study. Figure 2 shows the basic interface screenshots of miRToolsGallery. Users can view any published tools in miRToolsGallery, vote and rank them, and can also upload new tools (Submit tools tab, Figure 2) or submit an error report on a separate feedback page (Contact us tab, Figure 2).

**Figure 3.**
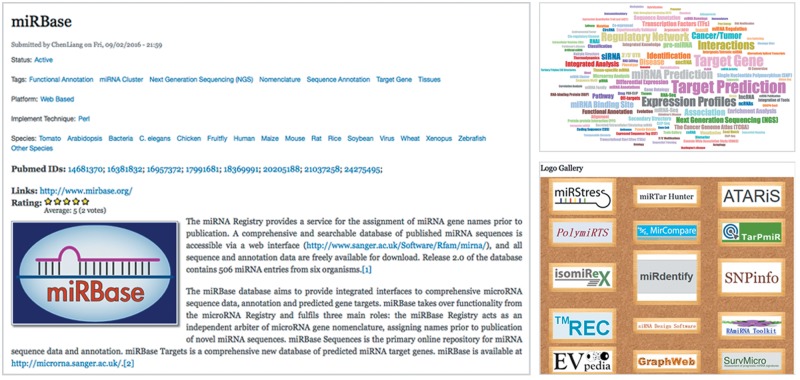
Screenshots of other important pages in miRToolsGallery. The left sub-image is an example of introduction of tools. Every tool in the database has the same information display structure based on Table 1. The top right panel shows a word cloud to illustrate the popular features of tools in the database. The bottom right panel shows the Logo Gallery which is a random list of tools with their logos in the database.

The user can search for tools by words or a phrase in the search sidebar or click the list of tools directly to obtain information about the tools. Every newly submitted tool will be promoted to the homepage (Shown in Figure 2) and basic statistics will be shown in the sidebar. The user can navigate the website by clicking the tabs (Figure 2) which contain the following options: (a) ‘Tutorials’ gives the user some practical cases and describes how the miRToolsGallery should be used; (b) ‘Tags Gallery’ shows the tags in the database as a word cloud and illustrates the term usage in miRToolsGallery (Figure 3); (c) ‘Logo Gallery’ randomly shows the logos of the tools on the page; (d) ‘Review Paper Gallery’ collects review papers which describe and compare miRNA tools; (e) ‘Submit Tools’ tab is a place for a user to contribute to the database and send new tools to the curator for updating miRToolsGallery and (f) ‘Contact Us’ tab provides users a way to interact with the administrator and send reports to improve the database.

**Figure 4.**
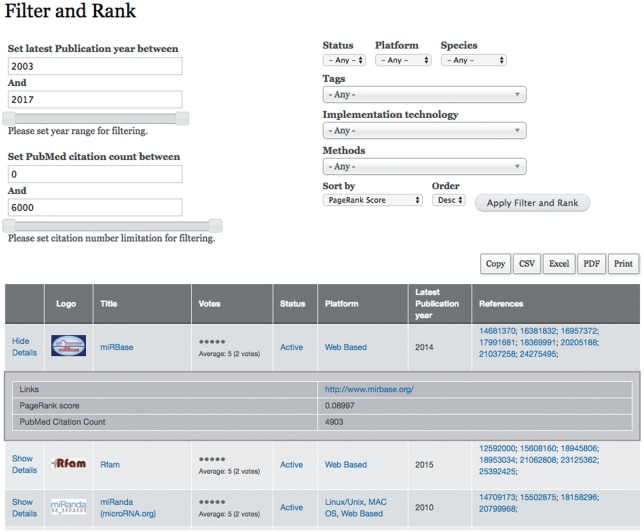
Screenshot of filter and rank page. The Filter and Rank tab is the most important point of entry of miRToolsGallery. At the top right, the selection list of filter and sort criteria are shown. All of the six tag groups are listed here as selection lists and a rank selection list follows. At the top left, two sliders are shown for filtering tools into a certain range of Latest Publication Year and Citation Count. After filter and rank selection has been performed, the tools table will list all the tools that meet the filter condition. The Logo, Title, Votes, Status, Platform, Latest Publication Year, References columns are shown in default. Links, PageRank score and PubMed citation count are listed in collapsible rows.

Because resources are diverse, and some are used more widely than others, a filter and rank tool has been implemented in the portal (Shown in Figure 4). miRToolsGallery gives users several options to query and filter which are based on taxonomy terms. The user can rank the tools in various ways, such as by PageRank score, latest publication year, publication number, citation count and vote. For example, if a user wants to find the most cited tools, he can choose the sort option ‘PubMed Citation Count.’ By clicking a tool, the user will see its detail information which is organized by the sections in Table 1. By clicking an option in the tags list, the user will directly get a catalogue of tools related to that tag.

**Table 1.** Sections in the tool’s information page used in miRToolsGallery

Section name	Description
Name of tool	Name of tools (If not available, title of paper is used).
Taxonomy tags	List of the available tags.
Article links	Link to PubMed (List all related references).
Tool links	Link to the homepage or source code (Any useful link could guide you to find the resources).
Vote	A five star scoring system is used.
Logo	Logo of the tool (If not available, a text of the tool’s name will be used instead).
Introduction	Abstract of paper extract from PubMed.
Reference	Link to more detail of literature.

### Comparison with other tools

NRDR, published in 2012 with the latest update in October 2014, is a collection of 140 ncRNA databases including 70 miRNA tools. It classifies the databases by RNA family, information source, information content and available search mechanisms (5). Tools4miRs is the first database focusing on miRNA tools integration and has currently gathered and categorized 198 tools (6). miRandb as a meta-database of web-based miRNA tools (7), classifies 188 tools into 8 classes and also provides a rank criterion called relative popularity which is extracted from third party commercial website traffic, statistics and analytics tool Alexa (http://www.alexa.com/siteinfo). OMICtools is an informative directory for multi-omic data analysis tool: if a search keyword such as miRNA or microRNA is provided, about 500 miRNA related tools out of ∼20,000 tools will be returned (22).

miRToolsGallery collects miRNA tools with a loose selection criteria, including not only tools designed for miRNA analysis, but also databases that can be applied for miRNA study. For example, Rfam which is the RNA families’ database can extract miRNA family information (23, 24). miRToolsGallery also provides two special computing related tags: ‘Implementation Technology’ and ‘Methods.’ ‘Implementation Technology’ is useful for bioinformaticians or biologists who want to find the source code written in their favourite programming language. ‘Methods’ can help researchers to find optimal computational methods to solve miRNA data analysis problems.

Table 2 shows a comparison between miRToolsGallery against other similar tools in various aspects. We excluded OMICtools from the comparison list since it is a general omic data analysis tool and not specific for miRNA. miRToolsGallery dominates over others in organizing and ranking the miRNA tools. The latest version of miRToolsGallery stores 970 tools and will be updated regularly in the future. Although miRandb has ranking function, it only collects web-based tools, so the tools rank is mainly based on the traffic of the website link. However, for some stand-alone software, a user might download the tool but not visit the link again, therefore rank by traffic of links will be biased towards frequently used web-based software. miRToolsGallery uses PageRank score of the tools citation network as the rank criteria, and the advantage is that it shows the influence of each tool in the miRNA tools community. In addition to PageRank score, there are other rank criteria. For example, latest publication year helps the user find the latest tools, PubMed citation count marks the well cited tools, publication count represents the maintenance activity and longevity of tools and average vote shows the rank in the users’ evaluation. The full list of tools with ranking scores is in Supplementary Table S1. Based on Pagerank score, the top 10 miRNA tools are miRBase (25), Rfam (23, 24), miRanda (26), MiRscan (27), TargetScan (28), miRNA–Target Gene Prediction at EMBL (29), PicTar (30), RNAhybrid (31), RNAz (32) and ViennaRNA (33). Not surprisingly, two miRNA sequence annotation registry databases are at the top followed by miRNA target prediction tools, then RNA secondary structure prediction tools.

**Table 2.** Comparison table with other databases

Tools	NRDR	Tools4miRs	miRandb	miRToolsGallery
RNA families	Non-coding RNA	miRNA	miRNA	miRNA, siRNA, piRNA
Tools type	Database, Web service	Database, Web service, Stand-alone	Database, Web service	Database, Web service, Stand-alone
Tools No.	140	198	188	970
Search^a^	Global	Partial	Global	Global
Rank	No	No	Yes	Yes
Filter	Yes	Yes	Yes	Yes
Tag	Yes	Yes	No	Yes
Vote	No	Yes	No	Yes
Category strategy^b^	One to Many	One to Many	One to One	One to Many
Latest update	October 2014	13 September 2017	Unknown	10 September 2017
Link	http://ncrnadatabases.org	http://tools4mirs.org	http://mirandb.ir	http://mirtoolsgallery.org

a Global: Search text in all the tools. Partial: Tools4miRs can only search those tools that are in the same category.

b One to Many: one tool could belong to different categories. One to One: one tool could only be classified into one category.

**Figure 5.**
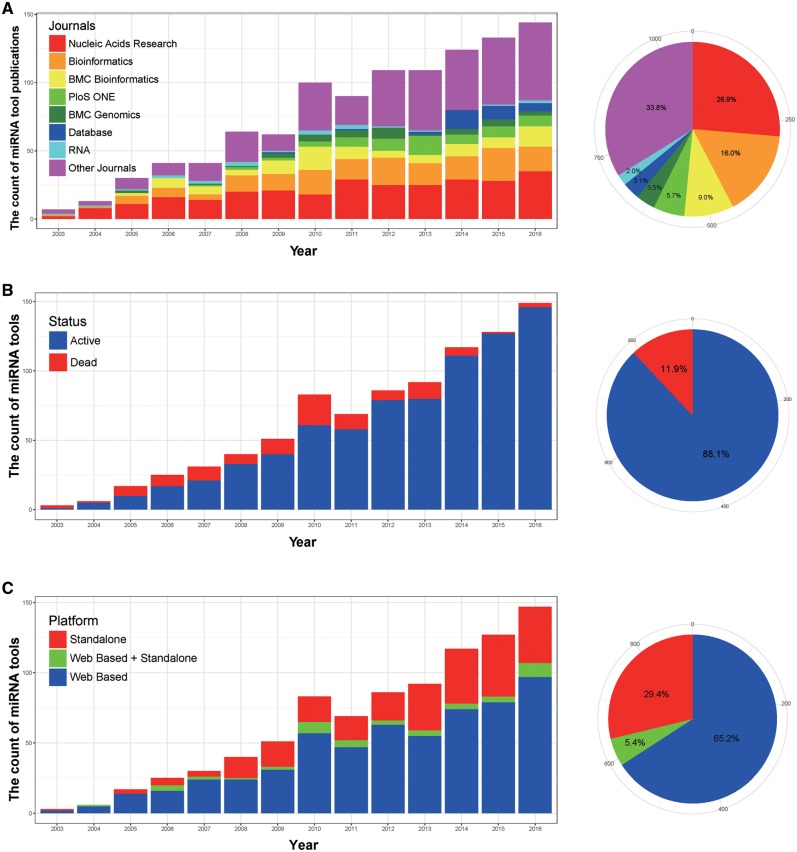
Basic statistics of miRToolsGallery. (**A**) Number of publications in different journals as a bar chart of tools by year. Each bar represents the number of publications describing miRNA tools in that year and the colours represent different journals. One-hundred-seventy-two journals were merged into ‘Other Journals,’ and a full list of journals with tool counts are reported in Supplementary Table S2. The pie-chart shows the percentage of total publications of miRNA tools for different journals. (**B**) Tool activity as a bar chart by year. Each bar represents the number of miRNA tools available in that year and the colour represents whether the tool was active or dead. The pie-chart shows the percentage of tools still available, and those that are inaccessible (dead). (**C**) The statistic of types of tool as a bar chart by year. Each bar represents the number of miRNA tools in that year, and web based, stand-alone or a blended type (web based + stand-alone) are labelled by three different colours. The pie-chart shows the percentage of those different types based on all the tools.

After mining the records in miRToolsGallery, we observed interesting trends from 2003 to 2016 that the number of publications describing bioinformatics tools rose rapidly from 2003 to 2010, then appeared to level off as shown in Figure 5A. The range of publications communicating these tools are highly varied overall, with tools published in 179 different journals, but surprisingly, the top 7 journals capture a high percentage (∼66%) of all publications. They are Nucleic Acids Research, Bioinformatics, BMC Bioinformatics, PloS ONE, BMC Genomics, Database and RNA ordered by tool count. The full list of journals with miRNA tool publication count is in Supplementary Table S2 (includes tools published in 2017). Tools of 108 out of 970 in miRToolsGallery are not active, as shown in Figure 5B. We merged Linux/Unix, MAC OS and Windows platforms into a stand-alone software catalogue to generate Figure 5C. From Figure 5C, we observed that web based tools are more popular than stand-alone tools by a high percentage (∼71%). There was only a small fraction (∼5.4%) of tools that offer stand-alone software and web services simultaneously. The prominent tool examples are TargetScan and RNAhybrid.

## Discussion

We have built a novel manually curated database, miRToolsGallery, for storing miRNA related analysis tools. As an increasing number of bioinformatics tools are being developed and published for miRNA analysis, it is becoming time consuming and laborious for researchers to find the appropriate tool for their study. For example, a laboratory bench scientist who may not be familiar with programming might want to find an easy to use web server with a friendly user interface. On the other hand, a computational biologist might want to find some miRNA feature datasets or annotations for their study, or might want to design a pipeline for automating a repetitive analysis. In this case, a candidate tool list and system for how to prioritize them is needed and miRToolsGallery is exactly an efficient resource integrator for finding the appropriate tools. miRToolsGallery can also ease the process of collecting available miRNA tools for the purposes of reviewing the field, or benchmarking tools for a specific application and finally release users from tedious Google searches. In addition, current miRNA bioinformatics resources, including the database, web service and source code etc. in miRToolsGallery could also help interested users to better understand the methodology and re-use it in new practical applications.

In comparison with NRDR, Tools4miRs, miRandb and OMICtools, miRToolsGallery has several advantages. First, miRToolsGallery includes significantly more miRNA related tools while still maintaining good organization. Second, miRToolsGallery is the only one that can rank the tools with a popularity based citation network. PageRank score is a good method to represent the popularity of each tool in the whole miRNA tools network. Also other rank criterions can help the user to select tools for other purposes. Third, compared with hierarchical classification systems, the tagging system is flexible and new tags will not affect older tags (34). Curators can involve more tags for the tools and this action can help the user to maximize the possibility to retrieve tools through relevant words. Fourth, miRToolsGallery is developed by applying Drupal 7, which provides an excellent platform and enough space for future upgrades.

Under continuous development and maintenance, miRToolsGallery will update regularly (e.g. for every 100 new tools or yearly). While miRToolsGallery is designed to store current miRNA bioinformatics research resources and tools comprehensively, it still has several limitations. Firstly, the current version of miRToolsGallery only collects those tools published and recorded in PubMed which does not record some other useful published miRNA tools. In the future, we may fix this by writing a new program to extract the citation network from the literature instead of using PubMed ID dependent methods. Secondly, checking the availability of resources is still performed by manual curation. We found many links to the tools that have been changed since the original published paper. Most of them provide an auto jump link to the new location, but for others, a deep Google search was needed to find the current website addresses. Our vision is that more and more researchers will find miRToolsGallery to be useful, and the feedback and updates from the whole miRNA community will contribute in keeping miRToolsGallery updated.

In the future, we plan to make this system more open, and build a miRNA tools community, for example, by opening the comment region to every user. Some tools will get a special allocation for backup of each version of the source code in case the original resource is abandoned or updated. Since miRToolsGallery was developed using Drupal 7, it is resident in a flexible and extendable framework. Thus, new rank strategies to sort the tools, new recommendation methods, or new catalogues are easy to add-in. Follow-up network analysis will also be implemented based on the tools-tags and tools-tools network in order to view relationships between tools. In summary, we constructed a database portal that researchers will find useful for analysing miRNA, and that may find other unexpected uses in the research of nucleic acids.

## Supplementary Material

Supplementary Data

Supplementary Table 1

Supplementary Table 2
