# Supplementary material for: miRToolsGallery: a tag-based and rankable microRNA bioinformatics resources database portal
Source: Database (Oxford). 2018 Feb 19;2018:bay004. doi: 10.1093/database/bay004 (PMC5819725; doi:10.1093/database/bay004)
Supplement: Supplementary Data [file bay004_supp_methods.docx]

# Custom scripts for miRToolsGallery

[Step 1: R code for extracting article information from PubMed 2](#_Toc500755220)

[Step 2: Perl code for parsed the XML output by Step 1 3](#_Toc500755221)

[Step 3: Perl code for constructing the network of tools base on Step 2 4](#_Toc500755222)

[Step 4: R code for PageRank the tools 5](#_Toc500755223)

## Step 1: R code for extracting article information from PubMed

# install.packages("rentrez")

library('rentrez')

##########################################################

# functions

##########################################################

## make dir

make.dir<-function(path){

if(file.exists(path)){

# print(paste(path," is exists!"))

return (F)

} else{

dir.create(path)

return (T)

}

}

isEmptyFile <- function( file ){

if( file.info(file)$size < 5){

return (T)

} else{

return (F)

}

}

## get references or cited paper PMID for input literature

get.refs.and.citedin <- function(PMID){

tryCatch({

hox_data <- entrez_link(db="pubmed", id = PMID, dbfrom="pubmed")

},

warning = function(w) {

print (paste(PMID,"->","Warning!\n",sep=""))

Sys.sleep(abs(rnorm(1)) + 10)

hox_data <- entrez_link(db="pubmed", id = PMID, dbfrom="pubmed")

}, error = function(e) {

print (paste(PMID,"->","Errors!\n",sep=""))

Sys.sleep(abs(rnorm(1)) + 15)

hox_data <- entrez_link(db="pubmed", id = PMID, dbfrom="pubmed")

}, finally = {

data <- list(citedin = hox_data$links$pubmed_pubmed_citedin , refs = hox_data$links$pubmed_pubmed_ref)

}

)

return (data)

}

get.info.xml <- function(PMID){

data <- entrez_fetch(db = "pubmed",

id = PMID,

rettype = "xml")

return (data)

}

# This R script could be running on different platform

# use place to choose the system

# place : Linux or Windows

place = "Linux"

if(place != "Linux"){

output.path = "path\\to\\output\\folder"

input.PMID.file = "path\\to\\PMID\\list\\file"

separator = "\\"

} else{

output.path = "path/to/output/folder"

input.PMID.file = "path/to/PMID/list/file"

separator = "/"

}

data <- read.table(file = input.PMID.file, header = F, sep="\t")

PMIDS <- unique(data$V1)

# showing the version we used

sink(paste(output.path,separator,"DB_build_version_and_Messages.log", sep=""),append = FALSE, type=c("output", "message"))

entrez_db_summary("pubmed")

###############################################

# download all the artical in the same xml

###############################################

count = length(PMIDS)

output.all.in.one.file = paste(output.path,separator,"all.in.one.", count, ".xml", sep="")

if(!file.exists(output.all.in.one.file)){

step = 200

start <- seq(1, ceiling(count/step)*step,by=step)

end <- seq(step,floor(count/step)*step, by=step)

end <- c(end, count)

xml.str = ""

for(i in c(1:length(start))){

subset.PMIDS = PMIDS[start[i]:end[i]]

data <- entrez_fetch(db = "pubmed",

id = subset.PMIDS,

rettype = "xml")

xml.str <- paste(xml.str,data,sep = "\n")

}

write(xml.str, file = output.all.in.one.file )

}

for( PMID in PMIDS){

dir.path <- paste(output.path,separator,"citations_references",separator,PMID,sep="")

if(make.dir(dir.path)){

}else{

print(paste(dir.path," is exists!"))

next

}

print(dir.path)

refs.citedin <- get.refs.and.citedin(PMID)

# write references

Sys.sleep(abs(rnorm(1)) + abs(rnorm(1)) + 5)

refs.file = paste(dir.path,separator,PMID,".refs",sep="")

write(refs.citedin$refs, file = refs.file)

# write citedin

citedin.file = paste(dir.path,separator,PMID,".citedin",sep="")

write(refs.citedin$citedin, file = citedin.file)

}

sink()

## Step 2: Perl code for parsed the XML output by Step 1

#!/usr/bin/perl

use String::Util qw(trim);

my $path = "path\\to\\Step1\\output\\folder";

my $XML_FILE = "path\\to\\Step1\\output\\folder\\all.in.one.xml";

my $COLLECTION_FILE = "path\\to\\input\\folder\\INPUT.DatabaseCollection.txt";

my $BLACKLIST_JOURNAL_FILE = "path\\to\\input\\folder\\INPUT.JournalBlacklist.txt";

my $BLACKLIST_YEAR_FILE = "path\\to\\input\\folder\\INPUT.PMID2YEARBlacklist.txt";

my $output_file = $XML_FILE.".parsed.txt";

my $output4R = $XML_FILE.".parsed.4.R.txt";

my ($tp1, $tp2) = initialHashs($COLLECTION_FILE);

my %PMID2NAME = %{$tp1};

my %PMID2LINKS = %{$tp2};

my %LongName2ShortName = %{initialJournalHash($BLACKLIST_JOURNAL_FILE)};

my %PMID2YEAR = %{initialPMID2YearHash($BLACKLIST_YEAR_FILE)};

open OFILE , ">".$output_file or die ("Sorry!\n");

open OFILE4R , ">".$output4R or die ("Sorry!\n");

my $xml = load_total_xml($XML_FILE);

# remove all enter "\n"

$xml =~ s/\n//g;

# parsed all articals

my @articals_xml = ($xml =~ /<PubmedArticle>(.*?)<\/PubmedArticle>/g);

my $num = @articals_xml;

print OFILE4R "PMID\tName\tTitle\tYear\tJournal\tISSN\tCitation count\n";

foreach my $artical_xml (@articals_xml){

my $PMID = get_PMID($artical_xml);

my $citedin_file = $path."\\".$PMID."\\".$PMID.".citedin";

my $refs_file = $path."\\".$PMID."\\".$PMID.".refs";

my @citedin_refs = @{get_PMID_list($citedin_file)};

my @refs = @{get_PMID_list($refs_file)};

my $title = get_paper_title($artical_xml);

my @keywords = @{get_keywords($artical_xml)};

my $article = get_article($artical_xml);

my @abstrcts = @{get_abstract($article)};

my $journal = get_journal($article);

my $journal_info = get_journal_info($journal, $PMID, \%PMID2YEAR, \%LongName2ShortName);

my $citation_num = 0;

if($citedin_refs[0] ne ""){

$citation_num = @citedin_refs;

}

my $name = $PMID2NAME{$PMID};

if($name eq ""){

$name = getToolName($title);

}

my $links = $PMID2LINKS{$PMID};

print OFILE $PMID."\t".$name."\t".$title."\t".$links."\t".join(";",@keywords)."\t".join("<br>",@abstrcts)."\t".$journal_info."\t".$citation_num."\t".join(";",@citedin_refs)."\t".join(";",@refs)."\n";

print OFILE4R $PMID."\t".$name."\t".$title."\t".$journal_info."\t".$citation_num."\n";

}

print "There are ".$num." articals in the xml!\n";

close OFILE;

close OFILE4R;

print "Done!\n";

sub initialJournalHash{

my $file = $_[0];

my %LongName2ShortName = ();

open FILE , "<".$file or die ("Sorry!($file)\n");

#remove header

readline(FILE);

while(<FILE>){

my @temp = split /\t/ , $_ ;

my $LongName = trim($temp[1]);

my $ShortName = trim($temp[0]);

if(defined $LongName2ShortName{$LongName}){

print "$LongName is duplicated!\n";

}

$LongName2ShortName{$LongName} = $ShortName;

}

close FILE;

return (\%LongName2ShortName);

}

sub initialPMID2YearHash{

my $file = $_[0];

my %PMID2YEAR = ();

open FILE , "<".$file or die ("Sorry!($file)\n");

readline(FILE);

while(<FILE>){

my @temp = split /\t/,trim($_);

my $pmid = $temp[0];

my $year = $temp[1];

$PMID2YEAR{$pmid} = $year;

}

close FILE;

return (\%PMID2YEAR);

}

sub initialHashs{

my $file = $_[0];

my %PMID2name = ();

my %PMID2links = ();

open FILE , "<".$file or die ("Sorry!($file)\n");

#remove header

readline(FILE);

while(<FILE>){

my @temp = split /\t/ , trim($_) ;

my $PMID = trim($temp[0]);

my $name = trim($temp[1]);

my $links = trim($temp[3]);

if(defined $PMID2name{$PMID}){

print "$PMID is duplicated!\n";

}

$PMID2name{$PMID} = $name;

$PMID2links{$PMID} = $links;

}

close FILE;

return (\%PMID2name,\%PMID2links);

}

sub load_total_xml{

my $file = $_[0];

open FILE , "<".$file or die ("Sorry can not open $file!\n");

local $/=undef;

my $xml = <FILE>;

close FILE;

return $xml;

}

sub get_PMID{

my $xml = $_[0];

my $PMID = "";

if( $xml =~ /<PMID.*?>(.*?)<\/PMID>/g){

$PMID =$1;

}

return $PMID;

}

sub get_article{

my $xml = $_[0];

my $article = "NULL";

if( $xml =~ /<Article.*?>(.*)<\/Article>/g ){

$article = $1;

}

return $article;

}

sub get_journal{

my $xml = $_[0];

my $journal = "NULL";

if( $xml =~ /<Journal>(.*)<\/Journal>/g ){

$journal = $1;

}

return $journal;

}

sub get_paper_title{

my $xml = $_[0];

my $title = "NULL";

if( $xml =~ /<ArticleTitle>(.*)<\/ArticleTitle>/g ){

$title = $1;

}

return $title;

}

sub getToolName{

my $title = $_[0];

my $name = "";

my @temp = split /:|\-{2}/,$title ;

if(@temp >= 2){

$name = $temp[0];

}

return $name;

}

sub get_abstract{

my $xml = $_[0];

my @abstrcts ;

my $list = "NULL";

if( $xml =~ /<Abstract.*?>(.*)<\/Abstract>/g ){

$list = $1;

}

while($list =~ /<AbstractText.*?>(.*?)<\/AbstractText>/g){

push @abstrcts, $1;

}

return \@abstrcts;

}

sub get_keywords{

my $xml = $_[0];

my @keywords ;

my $list = "NULL";

if( $xml =~ /<KeywordList.*?>(.*)<\/KeywordList>/g ){

$list = $1;

}

@keywords = ($list =~ /<Keyword.*?>(.*?)<\/Keyword>/g );

return \@keywords;

}

sub get_journal_info{

my $xml = $_[0];

my $pmid = $_[1];

my %PMID2YEAR = %{$_[2]};

my %LongName2ShortName = %{$_[3]};

my $ISSN = "NULL";

my $title = "NULL";

my $year = "NULL";

if( $xml =~ /<ISSN.*?>(.*)<\/ISSN>/g ){

$ISSN = $1;

}

if( $xml =~ /<Year>(.*)<\/Year>/g ){

$year = $1;

}

if( $xml =~ /<Title>(.*)<\/Title>/g ){

$title = $1;

}

if(defined $LongName2ShortName{$title}){

$title = $LongName2ShortName{$title};

}

if( $year eq "NULL"){

if(defined $PMID2YEAR{$pmid} ){

$year = $PMID2YEAR{$pmid};

} else{

print "Please put $pmid into blacklist (INPUT.PMID2YEARBlacklist.txt) !\n";

}

}

return $year."\t".$title."\t".$ISSN;

}

sub get_PMID_list{

my $file = $_[0];

my %PMID ;

open FILE , "<".$file or die ("Sorry can not open $file!\n");

while(<FILE>){

my $id = trim($_);

$PMID{$id} = $id;

}

close FILE;

my @PMIDS = keys %PMID ;

return \@PMIDS;

}

## Step 3: Perl code for constructing the network of tools base on Step 2

#!/usr/bin/perl

use String::Util qw(trim);

my $path = "path\\to\\Step1\\output\\folder";

my $PMID_FILE = "path\\to\\input\\folder\\INPUT.PMID.txt";

my $TOOLS_FILE = "path\\to\\input\\folder\\INPUT.DatabaseCollection20170909.txt";

my $PMID2YEAR_FILE = "path\\to\\Step1\\output\\folder\\all.in.one.xml.parsed.4.R.txt";

my $output_tool2pmids_file = "path\\to\\Step2\\output\\folder\\tool2pmids_and_citedinpmids.txt";

my $output_citedin_file = "path\\to\\Step2\\output\\folder\\citedin.network.txt";

my $output_refs_file = "path\\to\\Step2\\output\\folder\\refs.network.txt";

my $node_attribute_file = "path\\to\\Step2\\output\\folder\\node.attribute.txt";

my $output_paper_network_file = "path\\to\\Step2\\output\\folder\\refs.network.trimed.txt";

my $output_tool_paper_citation_network_file = "path\\to\\Step2\\output\\folder\\citation.network.trimed.txt";

my @PMIDS = @{get_PMID_list($PMID_FILE)};

print "There are ".@PMIDS." papers!\n" ;

my %TOOL_PMID = ();

# one tool could have many publications

# so one tool could have several PMIDS

my %TOOL2PMIDS = ();

%TOOL2PMIDS = %{initHashTOOL2PMIDS($TOOLS_FILE)};

%PMID2PMIDS = %{initHashPMID2PMIDS(\%TOOL2PMIDS)};

my %TOOL2CITEDINPMIDS = ();

my %PMID2YEAR = ();

%PMID2YEAR = %{initHashTOOLS2YEAR($PMID2YEAR_FILE)};

foreach my $id (@PMIDS){

$TOOL_PMID{$id} = "TOOL";

}

my %OTHER_PMID = ();

foreach my $tool (keys %TOOL2PMIDS){

# print "Parsing ".$tool."\n";

my @PMIDS = @{$TOOL2PMIDS{$tool}};

my ($tp1, $tp2) = get_Tool_PMIDS_list($path,\@PMIDS);

my %PMIDS_CITEDIN_TOTAL = %{$tp1};

my %PMIDS_REFS_TOTAL = %{$tp2};

my @PMIDS_CITEDIN = keys %PMIDS_CITEDIN_TOTAL;

my @PMIDS_REFS = keys %PMIDS_REFS_TOTAL;

$TOOL2CITEDINPMIDS{$tool} = \@PMIDS_CITEDIN;

}

open OFILE_CITEDIN , ">".$output_citedin_file or die ("Sorry!\n");

open OFILE_REFS , ">".$output_refs_file or die ("Sorry!\n");

open OFILE_NETWORK, ">".$output_paper_network_file or die ("Sorry!\n");

open OFILE_CITATION, ">".$output_tool_paper_citation_network_file or die ("Sorry!\n");

%CITATIONS = ();

foreach my $id (@PMIDS){

# print "Parsing ".$id."\n";

my $citedin_file = $path."\\".$id."\\".$id.".citedin";

my $refs_file = $path."\\".$id."\\".$id.".refs";

my @PMIDS_CITEDIN = @{get_PMID_list($citedin_file)};

my @PMIDS_REFS = @{get_PMID_list($refs_file)};

my $source = "";

my $target = "";

my $interaction = "";

foreach my $pmid (@PMIDS_CITEDIN){

$source = $pmid;

$target = $id ;

if( ! defined $TOOL_PMID{$pmid} ){

$OTHER_PMID{$pmid} = "OTHER";

}

if($pmid ne ""){

my $link = "0";

if(defined $TOOL_PMID{$pmid} ){

$link = "1";

######################################

$tp = $source."\t".$link."\t".$target;

$tp = renameEdge(\%PMID2PMIDS, $tp);

if( ! defined $CITATIONS{$tp} ){

$CITATIONS{$tp} = 1 ;

}

######################################

}

$tp = $source."\t".$link."\t".$target;

$tp = renameEdge(\%PMID2PMIDS, $tp);

print OFILE_CITEDIN $tp."\n";

}

}

foreach my $pmid (@PMIDS_REFS){

$source = $id;

$target = $pmid ;

if( ! defined $TOOL_PMID{$pmid}){

$OTHER_PMID{$pmid} = "OTHER";

}

if($pmid ne ""){

my $link = "0";

if(defined $TOOL_PMID{$pmid} ){

$link = "1";

$tp = $source."\t".$link."\t".$target;

$tp = renameEdge(\%PMID2PMIDS, $tp);

print OFILE_NETWORK $tp."\n";

######################################

$tp = $source."\t".$link."\t".$target;

$tp = renameEdge(\%PMID2PMIDS, $tp);

if( ! defined $CITATIONS{$tp} ){

$CITATIONS{$tp} = 1 ;

}

######################################

}

$tp = $source."\t".$link."\t".$target;

$tp = renameEdge(\%PMID2PMIDS, $tp);

print OFILE_REFS $tp."\n";

}

}

}

close OFILE_CITEDIN;

close OFILE_REFS;

close OFILE_NETWORK;

foreach my $citation( keys %CITATIONS ){

print OFILE_CITATION $citation."\n";

}

close OFILE_CITATION;

saveTOOL2PMIDS($output_tool2pmids_file,\%TOOL2PMIDS,\%TOOL2CITEDINPMIDS, \%PMID2YEAR);

open FILE , ">". $node_attribute_file or die ("Sorry!\n");

foreach my $id (keys %TOOL_PMID ){

print FILE $id."\t".$TOOL_PMID{$id}."\n";

}

foreach my $id (keys %OTHER_PMID ){

print FILE $id."\t".$OTHER_PMID{$id}."\n";

}

close FILE;

print "Done!\n";

sub renameEdge{

my $PMID2PMIDS = $_[0];

my $edge = $_[1];

my @temp = split /\t/, $edge ;

my $source = "";

if(! defined $PMID2PMIDS{$temp[0]} ){

$source = $temp[0];

} else {

$source = $PMID2PMIDS{$temp[0]};

}

my $link = $temp[1];

my $target = $PMID2PMIDS{$temp[2]};

if(! defined $PMID2PMIDS{$temp[2]} ){

$target = $temp[0];

} else {

$target = $PMID2PMIDS{$temp[2]};

}

my $newedge = "".$source."\t".$link."\t".$target;

return ($newedge);

}

sub initHashPMID2PMIDS{

my $TOOL2PMIDS = $_[0];

my %PMID2PMIDS = ();

foreach my $toolname ( keys %$TOOL2PMIDS ){

my $temp = $TOOL2PMIDS{$toolname} ;

my $name = join(";", @$temp);

foreach my $pmid ( @$temp ){

if( defined $PMID2PMIDS{$pmid} ){

print $pmid." is duplicated!\n" ;

}

$PMID2PMIDS{$pmid} = $name;

#print $pmid." -> ". $name."\n";

}

}

return (\%PMID2PMIDS);

}

sub get_Tool_PMIDS_list{

my $path = $_[0];

my @PMIDS = @{$_[1]};

my %PMIDS_CITEDIN_TOTAL = ();

my %PMIDS_REFS_TOTAL = ();

foreach my $id (@PMIDS){

my $citedin_file = $path."\\".$id."\\".$id.".citedin";

my $refs_file = $path."\\".$id."\\".$id.".refs";

my @PMIDS_CITEDIN = @{get_PMID_list($citedin_file)};

my @PMIDS_REFS = @{get_PMID_list($refs_file)};

foreach my $tp (@PMIDS_CITEDIN){

$PMIDS_CITEDIN_TOTAL{$tp} = $tp;

}

foreach my $tp (@PMIDS_REFS){

$PMIDS_REFS_TOTAL{$tp} = $tp;

}

}

return (\%PMIDS_CITEDIN_TOTAL,\%PMIDS_REFS_TOTAL);

}

sub get_PMID_list{

my $file = $_[0];

my %PMID = () ;

open FILE , "<".$file or die ("Sorry can not open $file!(fun get_PMID_list)\n");

while(<FILE>){

my $id = trim($_);

if($id eq ""){

# print "pmid is null string!\n";

} else{

$PMID{$id} = $id;

}

}

close FILE;

my @PMIDS = keys %PMID ;

return \@PMIDS;

}

sub initHashTOOLS2YEAR{

my $file = $_[0];

my %PMID2YEAR = ();

open FILE , "<".$file or die ("Sorry!($file)\n");

readline(FILE);

while(<FILE>){

my @temp = split /\t/,trim($_);

my $pmid = trim($temp[0]);

my $year = trim($temp[3]);

$PMID2YEAR{$pmid} = $year;

}

close FILE;

return (\%PMID2YEAR);

}

sub initHashTOOL2PMIDS{

my $file = $_[0];

my %TOOL2PMIDS = ();

open FILE , "<".$file or die ("Sorry can not open $file!(fun initHashTOOL2PMIDS)\n");

# remove headers

readline(FILE);

while(<FILE>){

my @temp = split /\t/,$_;

my $PMID = trim($temp[0]);

my $TOOL = trim($temp[1]);

if( $TOOL eq ""){ print $PMID."tool title is null!\n" };

if( defined $TOOL2PMIDS{$TOOL} ){

push $TOOL2PMIDS{$TOOL}, $PMID;

} else{

my @tp;

push @tp, $PMID;

$TOOL2PMIDS{$TOOL} = \@tp;

}

}

close FILE;

return (\%TOOL2PMIDS);

}

sub saveTOOL2PMIDS{

my $file = $_[0];

my %TOOL2PMIDS = %{$_[1]};

my %TOOL2CITEDINPMIDS = %{$_[2]};

my %PMID2YEAR = %{$_[3]};

open FILE, ">".$file or die ("Sorry can not write $file!\n");

# print headers

print FILE "Tool Name\tLatest Publication Year\tPublication Count\tPMIDS\tTotal Citation Count\tCitedin PMIDS\n";

my @tools = keys %TOOL2PMIDS;

foreach $tool (@tools){

my @temp = sort(@{$TOOL2PMIDS{$tool}});

# print "-->". join ("---",@temp) . ">".$temp[-1]."\n";

my $max_pmid = $temp[-1] ;

my $latest_pub_year = $PMID2YEAR{$max_pmid};

print FILE $tool."\t".$latest_pub_year."\t".@{$TOOL2PMIDS{$tool}}."\t". join(";",@{$TOOL2PMIDS{$tool}})."\t".

@{$TOOL2CITEDINPMIDS{$tool}}."\t".join(";", @{$TOOL2CITEDINPMIDS{$tool}})."\n";

}

close FILE;

print "$file have been saved!\n";

}

## Step 4: R code for PageRank the tools

# install.packages('igraph')

# install.packages('reshape')

library(igraph)

library(reshape)

network.files <- c('citedin.network.txt', 'refs.network.trimed.txt','refs.network.txt')

path = "path\\to\\Step2\\output\\folder"

for( file in network.files){

network.file <- paste(path, file, sep="")

output.file <- paste(path,file,".PageRanked.csv",sep="")

network <- read.table(network.file, header = F, sep="\t")

network <- data.frame(source.node = network$V1, target.node = network$V3)

vector.network <- as.vector(t(network))

g <- make_graph(as.character(vector.network) , directed = T)

pagerank.score <- page_rank(g)$vector

pagerank.score <- data.frame(node = names(pagerank.score), score = pagerank.score)

pagerank.score <- pagerank.score[order(pagerank.score$score, decreasing=T),]

write.csv(pagerank.score, file = output.file)

}
