# Supplementary material for: miRToolsGallery: a tag-based and rankable microRNA bioinformatics resources database portal
Source: Database (Oxford). 2018 Feb 19;2018:bay004. doi: 10.1093/database/bay004 (PMC5819725; doi:10.1093/database/bay004)
Supplement: Supplementary Table 1 [file bay004_supp_table_1.pdf]

Tool Name	Latest Publication Year	Publication Count	Total Citation Count	PageRank Score
miRBase	2014	8	4903	0.089971711
Rfam	2015	7	1872	0.083137364
miRanda (microRNA.org)	2010	4	2446	0.051662267
MiRscan	2003	2	597	0.051438779
TargetScan	2015	5	5972	0.050041484
miRNA - Target Gene Prediction at EMBL	2005	2	850	0.034150576
PicTar	2006	3	1798	0.031303389
RNAhybrid	2006	2	900	0.02416889
RNAz	2010	3	334	0.022668872
ViennaRNA	2015	5	1489	0.020935933
DIANA-TarBase	2015	4	650	0.017168313
smiRNAdb	2007	1	1250	0.009220509
PITA	2007	1	748	0.008658808
miRDeep	2012	2	638	0.008112443
miRecords	2009	1	407	0.007540016
DIANA-microT	2013	4	438	0.007082146
Bowtie	2009	1	5634	0.006218964
targetrank	2007	1	133	0.006041816
fRNAdb	2009	2	115	0.005964958
NONCODE	2016	6	350	0.005773178
miRTarBase	2016	3	613	0.005501763
TargetFinder	2010	3	897	0.005360648
miRDB	2016	5	497	0.005254933
Transterm	2009	2	44	0.004951214
miR2Disease	2009	1	293	0.004900643
miRNAMap	2008	2	156	0.004852097
SOAP	2009	2	1641	0.004766136
RNAxs	2008	1	86	0.004758056
ElMMo	2007	1	126	0.004478044
RNA22	2012	2	575	0.004433506
DIANA-miRGen	2016	3	159	0.004336136
starBase	2014	3	377	0.004274154

CMfinder	2006	1	118	0.004117806
Maq	2008	1	1105	0.003933423
ExoCarta	2016	4	403	0.003866034
miRanalyzer	2011	2	174	0.003501209
RNAdb	2007	2	116	0.003412174
miRGator	2013	3	114	0.003241712
SEQanswers	2012	2	31	0.002792323
PED	2014	3	25	0.002792323
miRU	2005	1	105	0.002728874
MicroInspector	2005	1	82	0.002697088
mirWIP	2008	1	93	0.002633642
HMDD	2014	2	278	0.002608325
DIANA-miRPath	2015	3	364	0.002572806
MPSS	2007	4	284	0.002519719
cre-siRNA	2007	1	146	0.002409417
Bioinformatics Links Directory	2012	8	46	0.002335886
Argonaute	2006	1	26	0.00206589
RNAmicro	2006	1	58	0.002045304
g:Profiler	2016	3	409	0.002026583
ASRP	2008	2	121	0.002024225
AHD	2011	2	29	0.001959525
maizeGDB	2016	4	100	0.001959525
MIMAS	2009	2	18	0.001959525
Laminin database	2014	2	4	0.001959525
RNAstructure	2016	3	300	0.001891049
siSearch	2004	1	36	0.001882992
ProMiR	2006	2	75	0.0018442
DIANA-LncBase	2016	2	75	0.001840769
DoRiNA	2015	2	82	0.001823025
BWA	2009	1	6318	0.001805722
RNAplex	2008	1	58	0.001798095
siDirect	2009	3	96	0.001768771
MicroTar	2006	1	24	0.001751575

mirclust	2009	1	19	0.001729239
TransmiR	2010	1	126	0.001647864
MIRZA	2013	1	42	0.001618446
SnoReport	2008	1	52	0.001606366
NPInter	2016	3	43	0.001586757
mirnasvm	2005	1	128	0.001549892
TargetSpy	2010	1	42	0.00148593
miRWalk	2015	2	531	0.001454392
MIReNA	2010	1	45	0.001430695
PolymiRTS	2014	3	138	0.001410779
psRNATarget	2011	2	377	0.001390829
miRmap	2013	2	43	0.001275883
RegRNA	2013	2	109	0.001270065
miRSel	2010	1	27	0.001252158
Antar	2011	1	31	0.001245782
MicroMUMMIE	2013	1	16	0.001236781
SVMicrO	2010	1	24	0.001230716
MIRZA-G	2015	1	7	0.001204228
Sfold	2014	4	166	0.001192462
miPred	2007	1	138	0.001190947
deep_sequencing	2010	1	56	0.001145903
MBSTAR	2015	1	1	0.001144634
mirTools	2013	2	61	0.001141264
TargetBoost	2005	1	31	0.001128818
GenMiR++	2007	1	155	0.001128775
deepBase	2016	3	75	0.001128144
PhenomiR	2012	2	71	0.001123091
siRecords	2009	2	23	0.001111082
MiRAlign	2005	1	83	0.001101067
siRNA Selection Server	2004	1	99	0.001096035
cellHTS	2010	2	120	0.001084918
miRExpress	2009	1	75	0.001081914
GenomeRNAi	2013	3	72	0.001077387

SigTerms	2008	1	60	0.001053198
miR-abela	2005	1	85	0.001048874
FlyRNAi	2012	2	58	0.001045286
DEQOR	2004	1	48	0.001033327
miTarget	2006	1	61	0.001033012
MAGIA	2012	2	82	0.00102064
NBmiRTar	2007	1	36	0.001009295
CircuitsDB	2011	2	51	0.000987094
LNCipedia	2015	3	154	0.000956451
TSGene	2016	2	127	0.000948225
IntaRNA	2014	2	146	0.000910714
SeqBuster	2016	3	49	0.000905051
DIANA-mirExTra	2016	2	34	0.000888233
UEA sRNA toolkit	2008	1	102	0.000883002
E-RNAi	2010	2	57	0.000873285
PARalyzer	2011	1	101	0.000868462
miRNASNP	2015	2	91	0.000867229
ChIPBase	2017	2	90	0.000858838
miRNAkey	2010	1	32	0.000854994
DSAP	2010	1	32	0.000850161
HuSiDa	2005	1	11	0.000844581
UCbase & miRfunc	2009	1	14	0.000833195
MMIA	2009	1	67	0.000816827
TargetMiner	2009	1	57	0.000816052
De Novo SVM Classification of Precursor Mic	2007	1	65	0.000815543
miRo	2009	1	38	0.000809363
soybean_mirna	2010	1	42	0.000793091
More complete gene silencing by fewer siRNAs	2007	1	19	0.000784818
Reliable prediction of Drosha processing sites i	2007	1	36	0.000782614
GraphWeb	2008	1	30	0.000772926
MISIM	2010	1	43	0.000767196
PMRD	2010	1	79	0.000766493
miRCancer	2013	1	55	0.000763491

EVpedia	2015	1	31	0.000763376
DSIR	2006	1	76	0.000753954
CluePedia	2013	1	67	0.000749171
piRNABank	2008	1	88	0.000748742
microPred	2009	1	57	0.00073854
MicroSNiPer	2010	1	43	0.000732759
FAME	2010	1	35	0.000721531
CORNA	2009	1	25	0.000720431
PASS	2009	1	51	0.000719285
miREnvironment	2011	1	19	0.000717858
SoyKB	2014	2	37	0.000716406
GeneSet2miRNA	2009	1	21	0.000704152
HLungDB	2010	1	18	0.000700334
Patrocles	2010	2	71	0.000697493
GenScript	2004	1	20	0.000687716
miRNA SNiPer	2013	2	26	0.000685373
segemehl	2009	1	148	0.000684077
Web MicroRNA Designer	2008	2	391	0.000683857
SNMNMF	2011	1	53	0.000675759
miRDeep*	2013	1	36	0.000673926
Empirical GO	2015	1	21	0.000670187
SignaLink	2013	2	50	0.000658752
miRNA Body Map	2011	1	34	0.000657761
miRNApath	2007	1	12	0.000656481
siDRM	2008	1	10	0.000655322
SHRiMP	2011	2	304	0.0006494
RNAither	2009	1	25	0.000649394
Tomato Functional Genomics Database	2011	2	57	0.000644766
PatMaN	2008	1	61	0.000644216
FLIGHT	2010	2	20	0.000642736
mirConnX	2011	1	49	0.000635813
miRGate	2017	2	10	0.000632329
dChip-GemiNI	2012	1	27	0.000629021

miTALOS	2016	2	19	0.000622995
sIR	2007	1	10	0.000614949
TAM	2010	1	42	0.000612001
SiteSifter	2010	2	221	0.000608367
MiRPara	2011	1	35	0.000605501
OligoWalk	2010	2	20	0.000603467
miRTar	2011	1	29	0.000600169
YM500	2017	3	28	0.000597438
dbDEMC	2017	2	26	0.000593095
SomamiR	2016	2	21	0.000592964
ShortStack	2016	2	38	0.000591686
miR2Subpath	2012	1	14	0.000591325
omiRas	2013	1	21	0.000590314
miRCode	2012	1	64	0.000589993
GeneCodis	2012	3	336	0.000588679
ncRNAdb	2007	1	18	0.000581193
SM2miR	2013	1	19	0.000574136
TSmiR	2014	1	26	0.00056837
MeT-DB	2015	1	7	0.000563196
Chipster	2011	1	66	0.000562101
lncRNASNP	2015	1	26	0.000561755
NCG	2016	5	83	0.000561745
CPSS	2017	2	15	0.000558782
isomiRex	2013	1	12	0.000557739
CleaveLand	2009	1	125	0.000557712
novoMiRank	2016	1	4	0.00055626
Database of isomirs	2010	1	104	0.000553792
The UEA sRNA workbench	2017	3	72	0.00054947
siRNA Design Software	2005	1	18	0.000548998
siRNArules	2006	1	10	0.000548688
TAPIR	2010	1	49	0.000548461
miRdSNP	2012	1	41	0.000547501
DARIO	2011	1	20	0.000547456

CAP-miRSeq	2014	1	25	0.000545244
MGD/MGI	2015	3	167	0.000543922
HCS-Analyzer	2012	1	4	0.000543768
BreastMark	2013	1	32	0.000543768
GeneSeer	2005	1	4	0.000543768
miRDeep-P	2011	1	54	0.000540364
MirSNP	2012	1	55	0.000539543
CSRDB	2007	1	39	0.000538804
miRNAminer	2008	1	21	0.000536796
NEXT-RNAi	2010	1	24	0.000527909
PNRD	2015	1	18	0.000519641
MiRFinder	2007	1	29	0.000517437
miRNEST	2014	2	17	0.000516488
TFmiR	2015	1	12	0.000514865
PIPmiR	2012	1	38	0.000513327
CID-miRNA	2008	1	21	0.000512076
LSD	2014	2	18	0.00051118
RCoS	2009	1	64	0.000510598
piRNA	2009	1	21	0.000509234
mirEX	2015	2	17	0.000504784
mirDIP	2011	1	66	0.000503904
A personalized microRNA microarray normaliz	2010	1	11	0.000502128
LoessM	2009	1	20	0.000502128
miREval	2013	2	20	0.000501025
MIR@NT@N	2011	1	28	0.000500513
RNAiDB	2004	1	29	0.000495086
Linc2GO	2013	1	23	0.000489613
AgiMicroRna	2011	1	46	0.000484062
VIRmiRNA	2014	1	12	0.000483589
MatureBayes	2010	1	26	0.000475151
BRB-ArrayTools	2008	1	22	0.000472973
mirna-corpora	2014	1	8	0.000472239
miRTarCLIP	2013	1	15	0.000470509

miROrtho	2009	1	29	0.000469726
PETfold & PETcofold	2011	4	48	0.000469206
wapRNA	2011	1	20	0.000469136
miRSystem	2012	1	51	0.000467117
workflow of integrating mRNA and miRNA ex	2011	1	11	0.000460488
CancerMiner	2013	1	49	0.000460332
siDesign	2009	1	20	0.00045994
TargetRNA	2014	2	79	0.000458784
miRvar	2011	1	11	0.000457318
IsomiRage	2014	1	15	0.000457129
miRDeathDB	2012	1	7	0.000456715
miRRim	2007	1	23	0.000454718
PhaseTank	2015	1	4	0.00045454
HHMMiR	2009	1	25	0.000454482
SyStemCell	2012	1	4	0.000454242
Lasso_miR	2011	1	35	0.000451632
SiDE	2005	1	10	0.000451611
SoymiRNet	2014	1	10	0.000450526
rice_build	2008	1	115	0.0004504
miren	2012	1	17	0.000449607
miRBase Tracker	2014	1	25	0.000448653
MIREAP	2015	2	25	0.000448486
miTEA	2013	1	13	0.000447402
Chimira	2015	1	10	0.000446231
MiClip	2014	1	8	0.000444279
PROmiRNA	2013	1	21	0.000441277
iMir	2013	1	15	0.000438871
miRDeepFinder	2012	1	87	0.000438322
multiMiR	2014	1	19	0.000436686
PuTmiR	2010	1	15	0.000434332
FatiGO +	2007	1	102	0.000433836
miRge	2015	1	7	0.00043134
MirZ	2009	1	35	0.00043017

CRSD	2006	1	22	0.000427176
SSCprofiler	2009	1	18	0.000422962
miRNAFold	2016	2	12	0.000422837
microTSS	2014	1	13	0.000422752
sRNAtoolbox	2015	1	23	0.000421799
dsCheck	2005	1	35	0.000420856
mirAct	2011	1	11	0.00042062
isomiRID	2013	1	7	0.000419392
OncomiRDB	2014	1	18	0.000419302
SpecificityServer	2008	1	6	0.000419217
AsiDesigner	2010	2	5	0.000419217
PASmiR	2013	1	9	0.000418848
MonarchBase	2013	1	15	0.000418848
Database of cattle candidate genes and genetic r	2009	1	28	0.000418848
PHDcleav	2013	1	8	0.000418848
sMBPLS	2012	1	19	0.000418848
miRiadne	2015	1	1	0.000418848
NetAge	2010	1	21	0.000418848
miRNA timeline	2012	1	18	0.000416354
TMREC	2015	1	3	0.000416194
ComiR	2013	2	45	0.000415748
AthaMap	2014	3	39	0.000415632
mirPub	2015	1	4	0.000415422
miR-host	2013	1	18	0.000415174
REA	2013	1	4	0.000415067
SMiR-NBI	2016	2	101	0.000413965
PmmR	2011	1	5	0.000412747
miRHrt	2010	1	9	0.000411998
mimiRNA	2010	1	29	0.000411686
STarMir	2016	2	17	0.000411532
CoGemiR	2008	1	11	0.000406988
miRFANs	2012	1	3	0.000406221
deepBlockAlign	2012	1	9	0.000405663

Target-align	2010	1	21	0.000403842
FANTOM4 EdgeExpressDB	2009	1	27	0.000403789
SeqTrimMap	2012	1	11	0.000401533
FlaiMapper	2015	1	5	0.000399741
shortran	2012	1	5	0.000399525
PmiRKB	2011	1	12	0.000399056
SAVoR	2012	1	7	0.000397957
MaturePred	2011	1	16	0.000397059
MiRdup	2013	1	11	0.00039549
mrSNP	2014	1	13	0.000395476
TALASSO	2012	1	19	0.00039319
CoRAL	2014	2	9	0.000392603
SFGD	2014	1	10	0.00039208
GraP	2015	1	5	0.00039208
DisTMGneT	2013	1	9	0.000391354
RIsearch	2012	1	17	0.000389498
mirsnpscore	2011	1	25	0.000389249
TargetScore	2014	1	7	0.000388612
PlantMiRNAPred	2011	1	18	0.000387816
UTRdb and UTRsite	2010	1	92	0.000387741
siRNAdb	2005	1	13	0.000387321
miRT	2012	1	8	0.000386902
HuntMi	2013	1	11	0.000386324
mirMark	2014	1	6	0.000386208
HOCTARdb	2011	1	16	0.000385401
dbSMR	2009	1	20	0.000385152
miRtest	2012	1	10	0.000384984
ceRDB	2012	1	20	0.000383628
ppt-miRBase	2011	1	7	0.000382934
ExprTargetDB	2010	1	36	0.000381081
RNAcentral	2017	3	47	0.000380572
MiRank	2008	1	20	0.000379764
rSNPBase	2014	1	20	0.000379374

MTide	2015	1	10	0.000379101
DAS	2005	1	5	0.000377209
miSolRNA	2010	1	1	0.000377209
RPdb	2015	1	1	0.000377209
KUPKB	2012	1	9	0.000377209
CellMiner	2012	1	98	0.000377209
miRandola	2012	1	52	0.000376073
MiRmat	2012	1	7	0.000375835
SylArray	2010	1	18	0.000374811
miRSponge	2015	1	9	0.000374433
ISMARA	2014	1	41	0.000372667
SeedSeq	2013	1	1	0.000372667
Pharmaco-miRs	2014	1	15	0.000372172
miRTex	2015	1	6	0.000372161
miR-BAG	2012	1	4	0.000371262
miRTrail	2012	1	12	0.000371238
A combinatorial approach to determine the cont	2009	1	2	0.000370858
ExcellmiRDB	2015	1	1	0.000370593
self-containment index calculation	2008	1	9	0.000369903
miRegulome	2015	1	4	0.000369645
sPARTA	2014	1	10	0.000369212
PEMDAM	2014	1	7	0.000369141
PAREsnip	2012	1	30	0.000368645
miREvo	2012	1	30	0.000367851
mRTP	2007	1	12	0.000367258
miRNACon	2014	1	18	0.000366028
miRror-Suite	2014	3	20	0.000364918
MicroPC	2009	1	8	0.000364226
Circ2Traits	2013	1	29	0.000363754
miRiam	2010	1	15	0.000363382
cWords	2013	1	6	0.000363329
miRMAP (Visualization)	2014	1	6	0.000361978
miRFam	2011	1	9	0.000361742

E-miR	2010	1	31	0.000361608
Vir-Mir db	2008	1	25	0.000360848
CCDB	2011	1	20	0.000360553
Oasis	2015	1	4	0.000359958
dPORE	2011	1	13	0.000359898
microTranspoGene	2008	1	33	0.000359843
SoMART	2012	1	22	0.000359209
miRMaid	2010	1	6	0.000359143
GAMUT	2014	1	3	0.000358332
ISRNA	2014	1	5	0.000357014
RFRCDB-siRNA	2007	1	11	0.000356389
RPASuite	2015	1	4	0.000356389
AltAnalyze and DomainGraph	2010	1	80	0.000356389
BioVLAB-MMIA-NGS	2015	1	4	0.000356132
miRspring	2013	1	7	0.00035592
ncRNAimprint	2010	1	14	0.000355714
TissueAtlas	2016	2	23	0.000354131
Xenbase	2015	2	43	0.000354131
HDMP	2013	1	23	0.000353967
tasiRNAdb	2014	1	7	0.000353713
RISCbinder	2009	1	14	0.000353266
MapMi	2010	1	22	0.000353125
targetHub	2013	1	4	0.000352919
cGRNB	2013	1	3	0.00035256
PsRobot	2012	1	49	0.000352175
SmiRN-AD	2014	1	6	0.000349878
Mirsynergy	2014	1	12	0.000349629
HMED	2014	1	7	0.000348399
MultiMiTar	2011	1	11	0.000348005
DeAnnIso	2016	1	2	0.000347871
MIRPIPE	2014	1	6	0.000347871
LncRBase	2014	1	7	0.000347587
M@IA	2008	1	4	0.000347108

ViTa	2007	1	27	0.000346693
MAGI	2014	1	3	0.000344923
miR2GO	2015	1	4	0.000344634
NorahDesk	2012	1	4	0.00034411
DualTargeting	2010	1	7	0.000343897
RNAimmuno	2012	1	11	0.000343897
microPIR	2014	2	14	0.000343833
mdgsa	2016	1	3	0.000342112
CMP	2011	1	10	0.00034175
vHoT	2012	1	6	0.000340373
Discriminant	2014	1	8	0.000339787
NOVOMIR	2010	1	5	0.000339663
mrsFAST	2014	2	111	0.000339619
miRNA-regulome	2015	1	3	0.000339501
miR-isomiRExp	2016	1	9	0.000339354
miRIAD	2014	1	10	0.000339039
MiRonTop	2010	1	17	0.000338593
PMTED	2013	1	7	0.000338098
p-TAREF	2011	1	7	0.000337842
eRNA	2014	1	8	0.000337803
ViRBase	2015	1	18	0.000337304
TAREF	2010	1	10	0.000337153
TUMIR	2013	1	3	0.000337118
S-MED	2010	1	28	0.000336077
TargetCompare	2014	1	3	0.000335569
GREENC	2016	1	7	0.000335569
MVDA	2015	1	4	0.000335569
CANTATAdb	2016	1	4	0.000335569
miRiaD (Text Mining Tool)	2016	1	1	0.000335569
MiRTif	2008	1	21	0.000335222
mirnaTA	2014	1	3	0.000334553
PMirP	2010	1	9	0.000334205
tfmirloop	2012	1	7	0.000333674

CyTargetLinker	2013	1	16	0.000333045
SITPR	2014	1	10	0.000333045
Small RNA Workbench	2013	1	6	0.00033285
miRvestigator	2011	1	15	0.000332539
HeteroMirPred	2013	1	10	0.000332517
rip	2010	1	10	0.000331912
Comparative Sequencing of Plant Small RNAs	2014	2	39	0.00032962
MIPS PlantsDB	2013	2	63	0.00032962
piRNA cluster database	2016	1	4	0.00032962
HPVbase	2015	1	6	0.00032962
siRNAmod	2016	1	4	0.00032962
Lists2Networks	2010	1	25	0.00032962
BraMRs	2012	1	12	0.00032962
miR-PREFeR	2014	1	10	0.00032962
CHNmiRD	2016	1	4	0.00032962
DISMIRA	2015	1	3	0.00032962
recit	2009	1	7	0.000329261
Psmir	2016	1	2	0.000329116
miRTarVis	2015	1	2	0.000328247
lncRNAMap	2014	1	7	0.000328035
microT-ANN	2011	1	5	0.000326938
mESAdb	2011	1	5	0.000326934
ncPRO-seq	2012	1	20	0.000326265
mirExplorer	2011	1	9	0.000326246
rnaanalys	2015	2	4	0.000326105
mircisreg	2008	1	4	0.000325196
CrossHub	2016	1	6	0.000325159
MMiRNA-Tar	2015	1	2	0.000325159
lnCeDB	2014	1	16	0.000325159
GUUGle	2006	1	13	0.000324026
mirna_target	2010	1	10	0.000324007
treebic	2011	1	2	0.0003219
mirTarPri	2013	1	3	0.000321689

miRNA-SNiPer	2015	1	3	0.000321689
ZooMir	2010	1	19	0.000321689
ncRNAclassifier	2012	1	4	0.000321619
Bioinformatics Resource Manager	2012	2	26	0.000321234
miRD	2011	1	7	0.00032116
RepTar	2011	1	14	0.000320941
APADB	2014	1	8	0.000320803
ActMiR	2016	1	5	0.000320462
ToppMiR	2014	1	5	0.000319145
GrapeMiRNA	2009	1	2	0.000319015
RBP-Var	2016	1	1	0.000318913
RMBase	2016	1	10	0.000318913
MiRduplexSVM	2015	1	3	0.000318913
miRNAprediction	2012	1	4	0.000318913
ifmda	2016	1	31	0.000316641
isomiR-SEA	2016	1	1	0.000316641
miRPlant	2014	1	9	0.000316641
ProMISe	2014	1	13	0.000316641
StarScan	2015	1	4	0.000316641
FARNA	2017	1	2	0.000316641
miRNeye	2010	1	59	0.000316081
miReg	2010	1	3	0.000314875
miRdentify	2014	1	4	0.000314749
PlantMirnaT	2015	1	3	0.000314749
miRHiC	2013	1	1	0.000314749
NRDR	2012	1	5	0.000314749
HomoTarget	2013	1	4	0.000314749
RandA	2012	1	5	0.000314653
miRPD	2014	1	14	0.000313941
tsmti	2014	1	5	0.000313938
Screensaver	2010	1	12	0.000313147
MiRNA GO Annotation Manual	2016	1	3	0.000312957
sRNAMap	2009	1	29	0.000312957

Cupid	2015	1	14	0.000312596
Pan-ceRNADB	2015	1	13	0.000312596
Geoseq	2010	1	3	0.000312108
PARma	2013	1	13	0.000311774
miReader	2013	1	5	0.000311774
SolmiRNA	2011	1	11	0.000311774
miRTour	2011	1	4	0.000311774
CLIPSeqTools	2016	1	5	0.000311774
Mirinho	2015	1	1	0.000311044
miRBoost	2015	1	2	0.000311044
HNOCDB	2012	1	5	0.000310585
mirSOM	2011	1	8	0.000310585
HOCCLUS2	2014	1	9	0.000310585
MIDP	2015	1	7	0.000310585
MISIS	2016	2	7	0.000310585
miRTCat	2013	1	6	0.000310254
iMcRNA	2015	1	43	0.000309544
miRNA-dis	2015	1	14	0.000309544
targetS	2014	1	7	0.000309544
iMiRNA-PseDPC	2016	1	27	0.000309544
miRNA-deKmer	2015	1	25	0.000309544
MNDR	2013	1	21	0.000309544
SplamiR	2011	1	2	0.000309209
myMIR	2011	1	8	0.00030832
EpimiR	2014	1	5	0.000307809
ERISdb	2013	1	9	0.000306472
iSRAP	2015	1	2	0.000306421
miRModule	2015	1	2	0.000306421
PROGmiR	2012	1	9	0.00030602
SurvMicro	2014	1	9	0.00030602
DASHR	2016	1	3	0.00030602
CancerNet	2015	1	2	0.00030602
miSEA	2015	1	2	0.000305826

Omics Pipe	2015	1	13	0.000305826
MirID	2013	1	2	0.000305285
Raccess	2011	1	13	0.000302257
HumanViCe	2014	1	2	0.000293929
ComiRNet	2015	1	1	0.000293929
miRLAB	2015	1	2	0.000293929
EvoRSR	2009	1	0	0.000293929
MiRComb	2016	1	1	0.000293929
GeneACT	2006	1	7	0.000293929
miR2Gene	2011	1	5	0.000293929
BCmicrO	2012	1	5	0.000293929
MiRNATIP	2016	1	0	0.000293929
SIPHT	2008	1	62	0.000293929
PerM	2009	1	36	0.000293929
mirCoX	2013	1	2	0.000293929
plantDARIO	2014	1	2	0.000293929
VetBioBase	2014	1	4	0.000293929
hLGDB	2013	1	4	0.000293929
TMMN	2015	1	4	0.000293929
Mirnacle	2016	1	1	0.000293929
LimiTT	2016	1	0	0.000293929
sydSeq	2015	1	1	0.000293929
ARN (Adipogenesis)	2016	2	0	0.000293929
SoyFN	2014	1	1	0.000293929
BCIP	2017	1	0	0.000293929
CyTRANSFINDER	2016	1	1	0.000293929
isomiR-Benchmark	2017	1	0	0.000293929
FARE-CAFE	2015	1	1	0.000293929
PDbase	2009	1	2	0.000293929
PGS	2014	1	2	0.000293929
CSCdb	2016	1	0	0.000293929
MITHrIL	2016	1	0	0.000293929
SparseMFEFold	2016	1	0	0.000293929

TargetExpress	2016	1	2	0.000293929
PmiRExAt	2016	1	0	0.000293929
LNCediting	2017	1	2	0.000293929
ODIN-bc2015-miRNA	2017	1	0	0.000293929
mirMeta	2016	1	0	0.000293929
PC-TraFF	2015	1	2	0.000293929
UP-TORR	2013	1	17	0.000293929
ncPred	2014	1	5	0.000293929
miRAS	2007	1	1	0.000293929
miR-Synth	2014	1	4	0.000293929
expmicro	2010	1	5	0.000293929
isomiR2Function	2017	1	0	0.000293929
SZGR	2010	1	42	0.000293929
SpidermiR	2017	1	0	0.000293929
RiboSubstrates	2006	1	6	0.000293929
MixMir	2014	1	4	0.000293929
GeneHub-GEPIS	2007	1	11	0.000293929
MiRAuto	2013	1	0	0.000293929
miARma-Seq	2016	1	4	0.000293929
GEAR	2017	1	0	0.000293929
XTalkDB	2017	1	0	0.000293929
unitas	2017	1	0	0.000293929
miRCluster	2012	1	2	0.000293929
CircNet	2016	1	11	0.000293929
A web-based bioinformatics interface applied tc	2012	1	10	0.000293929
ProteoMirExpress	2013	1	0	0.000293929
ProNet	2010	1	14	0.000293929
PsRNA	2010	1	1	0.000293929
CSZ	2014	1	5	0.000293929
miRnalyze	2017	1	0	0.000293929
Tailor	2015	1	3	0.000293929
MirGeneDB	2015	1	23	0.000293929
IntNetLncSim	2016	1	3	0.000293929

MirAncestar	2017	1	0	0.000293929
Avishkar	2015	1	2	0.000293929
ChroMoS	2013	1	16	0.000293929
LBSizeCleav	2016	1	2	0.000293929
DsTRD	2016	1	2	0.000293929
miRPathDB	2017	1	3	0.000293929
SNPinfo	2009	1	194	0.000293929
Infinity	2016	1	1	0.000293929
RDMAS	2006	1	15	0.000293929
PEpiD	2013	1	5	0.000293929
Ginger Est	2015	1	0	0.000293929
ncRNA-DB	2014	1	4	0.000293929
AtmiRNET	2015	1	4	0.000293929
Orchidstra	2017	2	10	0.000293929
ptRNApred	2014	1	1	0.000293929
fat_deposition	2013	1	11	0.000293929
ImiRP	2016	1	1	0.000293929
Ortho2ExpressMatrix	2011	1	1	0.000293929
GBM-BioDP	2014	1	15	0.000293929
Pancreatic Cancer Database	2014	1	10	0.000293929
MagiCMicroRna	2015	1	1	0.000293929
RNAi Codex	2006	1	17	0.000293929
ccNET	2017	1	3	0.000293929
GenomeTraFac	2007	1	13	0.000293929
microRNAviewer	2012	1	17	0.000293929
ChiloDB	2014	1	3	0.000293929
canEvolve	2013	1	10	0.000293929
Exo-miRExplorer	2017	1	1	0.000293929
CellBase	2012	1	11	0.000293929
CREAM	2014	1	1	0.000293929
NSDNA	2017	1	0	0.000293929
miRsig	2017	1	1	0.000293929
ViralmiR	2015	1	1	0.000293929

MysiRNA-designer	2011	1	7	0.000293929
BUFET	2017	1	0	0.000293929
ARN (Autophagy)	2015	1	6	0.000293929
Pipeline to analyze Illumina reads	2011	1	8	0.000293929
SMEpred workbench	2016	1	1	0.000293929
MixDTrees	2007	1	5	0.000293929
Listeriomics	2017	1	1	0.000293929
PceRBase	2017	1	1	0.000293929
RIDDLE	2012	1	10	0.000293929
GPA	2015	1	0	0.000293929
SEED	2011	1	15	0.000293929
gbCRC	2016	1	1	0.000293929
P-SAMS	2016	1	3	0.000293929
miRVine	2015	1	5	0.000293929
miRTar2GO	2017	1	0	0.000293929
SMIRP	2015	1	1	0.000293929
Structuring osteosarcoma knowledge	2014	1	8	0.000293929
ZIKV-CDB	2016	1	1	0.000293929
netClass	2014	2	8	0.000293929
miRcomp	2016	1	2	0.000293929
miREC	2015	1	1	0.000293929
miRTar Hunter	2013	1	2	0.000293929
MmPalateMiRNA	2013	1	3	0.000293929
miRNA_code	2009	1	4	0.000293929
Automatic learning of pre-miRNAs from differe	2016	1	1	0.000293929
PCBC	2017	1	0	0.000293929
DroID	2011	1	74	0.000293929
miRLocator	2015	1	1	0.000293929
VARIANT	2012	1	22	0.000293929
PHANTOM	2013	1	5	0.000293929
imRNA	2016	1	1	0.000293929
PeTMbase	2016	1	4	0.000293929
siVirus	2006	1	14	0.000293929

PPImiRFS	2015	1	1	0.000293929
OMICtools	2014	1	17	0.000293929
MtiBase	2015	1	5	0.000293929
SMiRK	2015	1	0	0.000293929
WSNF	2016	1	0	0.000293929
SHARAKU	2016	1	0	0.000293929
BiTargeting	2010	1	7	0.000293929
BCGSC miRNA Profiling Pipeline	2016	1	7	0.000293929
iNMF	2016	1	2	0.000293929
MCMG	2013	1	7	0.000293929
PAGED	2012	1	8	0.000293929
GARNET	2011	1	3	0.000293929
rVarBase	2016	1	6	0.000293929
IBRel	2017	1	0	0.000293929
SorghumFDB	2016	1	2	0.000293929
SubpathwayGMir	2015	1	2	0.000293929
miRNA Digger	2016	1	0	0.000293929
miRBShunter	2017	1	1	0.000293929
T-REX	2010	1	11	0.000293929
RScan	2007	1	1	0.000293929
RiceATM	2016	1	0	0.000293929
miRTargetLink	2016	1	5	0.000293929
Ebbie	2006	1	3	0.000293929
MirStress	2013	1	5	0.000293929
SePIA	2016	1	0	0.000293929
BmncRNAdb	2016	1	1	0.000293929
rna-wl	2010	1	1	0.000293929
COGERE	2014	1	4	0.000293929
ToppCluster	2010	1	61	0.000293929
Centratliy-based Pathway Enrichment	2012	1	13	0.000293929
LeARN	2008	1	7	0.000293929
HiTSeekR	2016	1	0	0.000293929
pssRNAMiner	2008	1	9	0.000293929

Seten	2017	1	0	0.000293929
miSTAR	2017	1	1	0.000293929
BatchGenAna	2009	1	0	0.000293929
SBM	2008	1	6	0.000293929
OncomiRdbB	2014	1	2	0.000293929
TRiP	2012	1	34	0.000293929
BBBomics	2016	1	1	0.000293929
DMD	2015	1	6	0.000293929
BiLTR	2015	1	3	0.000293929
si-shRNA Selector	2010	1	17	0.000293929
SubmiRine	2015	1	1	0.000293929
Mi-DISCOVERER	2010	1	0	0.000293929
RAIN	2017	1	0	0.000293929
DevMouse	2014	1	0	0.000293929
STarMirDB	2016	1	0	0.000293929
MMiRNA-Viewer	2016	1	0	0.000293929
fast-db	2007	1	23	0.000293929
MinDist	2012	1	0	0.000293929
ReNE	2014	1	3	0.000293929
BioM2MetDisease	2017	1	0	0.000293929
RAmiRNA	2012	1	0	0.000293929
C-mii	2012	1	5	0.000293929
RNA-Seq Viewer	2017	1	0	0.000293929
miRNALasso	2015	1	3	0.000293929
Vicinal	2014	1	5	0.000293929
BeadSme	2011	1	5	0.000293929
targetrunningsum	2015	1	2	0.000293929
GermlncRNA	2015	1	5	0.000293929
iSubgraph	2013	1	1	0.000293929
miratlas	2017	1	0	0.000293929
miRLiN	2016	1	1	0.000293929
miRquant	2016	2	19	0.000293929
iMiRNA-SSF	2016	1	23	0.000293929

TarPmiR	2016	1	0	0.000293929
MicroLive	2012	1	2	0.000293929
chimiRic	2016	1	1	0.000293929
MiRSEA	2016	1	0	0.000293929
miFRame	2015	1	1	0.000293929
siSPOTR	2013	1	12	0.000293929
MiREN	2016	1	2	0.000293929
mirPRo	2015	1	1	0.000293929
Ssa miRNAs DB	2012	1	3	0.000293929
AGD	2009	1	14	0.000293929
MREdictor	2013	1	7	0.000293929
CSmiRTar	2017	1	0	0.000293929
TGRD	2014	1	10	0.000293929
CKDdb	2017	1	0	0.000293929
TriplexRna	2014	1	8	0.000293929
miRMaster	2017	1	0	0.000293929
HTSmix	2011	1	4	0.000293929
AnnoLnc	2016	1	0	0.000293929
RENATO	2012	1	7	0.000293929
ChemiRs	2016	1	1	0.000293929
imiRTP	2012	1	7	0.000293929
plateletomics	2014	1	29	0.000293929
SimiRa	2015	1	2	0.000293929
SpecificDB	2007	1	0	0.000293929
miRA	2015	1	4	0.000293929
LMMEL-miR-miner	2016	1	0	0.000293929
PMF NETWORK MODEL	2016	1	1	0.000293929
pseudoMap	2013	1	1	0.000293929
RCDB	2012	1	11	0.000293929
Antagomirbase	2011	1	1	0.000293929
USAGP	2010	1	25	0.000293929
RNAdualPF	2016	1	1	0.000293929
UQAM Wheat microRNA Portal	2015	1	2	0.000293929

IGDB.NSCLC	2012	1	6	0.000293929
Cepred	2009	1	19	0.000293929
OvMark	2014	1	3	0.000293929
MD-SeeGH	2008	1	16	0.000293929
miRMOD	2015	1	0	0.000293929
VAN	2013	1	0	0.000293929
contextMMIA	2017	1	0	0.000293929
RNALOSS	2005	1	5	0.000293929
iDeep	2017	1	0	0.000293929
miRSeq	2014	1	5	0.000293929
SAMMate	2011	1	27	0.000293929
TTS mapping	2009	1	9	0.000293929
ENViz	2015	1	1	0.000293929
SbacHTS	2013	1	1	0.000293929
C2Analyzer	2014	1	0	0.000293929
CARD	2016	1	2	0.000293929
IntmiR	2011	1	0	0.000293929
ptrguide	2012	1	4	0.000293929
QuickMIRSeq	2017	1	0	0.000293929
SpermBase	2016	1	0	0.000293929
genYsis Toolbox	2013	1	1	0.000293929
RNA-CODE	2013	1	4	0.000293929
POSTAR	2017	1	0	0.000293929
miRNAsong	2016	1	1	0.000293929
spongeScan	2016	1	3	0.000293929
mythology	2015	1	3	0.000293929
CircInteractome	2016	1	10	0.000293929
MIRNA-DISTILLER	2011	1	0	0.000293929
ExiMiR	2014	1	1	0.000293929
mirdba	2013	1	3	0.000293929
RegNetwork	2015	1	12	0.000293929
GiSAO.db	2011	1	0	0.000293929
T2DiACoD	2017	1	0	0.000293929

H-RVM	2013	1	1	0.000293929
miEAA	2016	1	3	0.000293929
OCDB	2015	1	0	0.000293929
PACCMIT/PACCMIT-CDS	2015	1	1	0.000293929
CrossLink	2006	1	0	0.000293929
miREE	2011	1	3	0.000293929
TPS	2017	1	1	0.000293929
mBISON	2015	1	0	0.000293929
UTRome.org	2008	1	10	0.000293929
BioProfiling	2011	1	40	0.000293929
miR-EdiTar	2012	1	6	0.000293929
miRNet	2016	1	4	0.000293929
icTAIR	2017	1	0	0.000293929
miRVaS	2016	1	3	0.000293929
ATARiS	2013	1	47	0
ed_scan	2003	1	6	0
mirtronPred	2012	1	9	0
RespCanDB	2017	1	0	0
PlantcircBase	2017	1	2	0
miR_Path	2015	1	5	0
SIM	2016	1	1	0
MSbind	2014	1	1	0
MicRooN	2015	1	0	0
BioVLAB-MMIA	2012	1	4	0
PHMMTSs	2005	1	9	0
YamiPred	2015	1	0	0
1-CMDb	2017	1	0	0
miRmine	2017	1	1	0
OmniSearch	2016	1	2	0
MicroTarget	2017	1	0	0
Tools4miRs	2016	1	0	0
TopKLists	2015	1	1	0
Functional interpretation of microRNA-mRNA	2014	1	0	0

PheLiM	2017	1	0	0
DSTHO	2006	1	2	0
miRQuest	2016	1	0	0
CePa	2013	1	4	0
Exp. Verified microRNA-Target	2013	1	2	0
DynaMod	2010	1	4	0
Greglist	2008	1	19	0
ShrinkBayes	2014	1	2	0
GenoSkyline	2016	1	8	0
mirnaDetect	2013	1	3	0
MotifMap-RNA	2017	1	0	0
GO-Elite	2012	1	67	0
deepboost	2017	1	0	0
OligoFaktory	2006	1	5	0
EGs	2008	1	0	0
FMIGS	2017	1	0	0
ORCA	2015	1	0	0
BiCliques Merging	2016	1	0	0
TmiRUSite and TmiROSite scripts	2014	1	1	0
miRNAmeConverter	2017	1	1	0
MDTE DB	2015	1	0	0
MiRTDL	2016	1	0	0
PACdb	2010	1	29	0
RDDpred	2016	1	2	0
birta	2012	1	11	0
microDoR	2012	1	4	0
SCLC	2016	1	6	0
MicroRazerS	2010	1	14	0
DINGO	2015	1	4	0
iBFE	2015	1	0	0
ARTS	2006	2	47	0
miMsg	2013	1	0	0
NRSE	2006	1	2	0

IDT SciTools	2008	1	38	0
SARS	2013	1	1	0
MetaMirClust	2016	1	1	0
G-DOC	2013	1	2	0
sRNATarget	2008	1	12	0
MIRAGAA	2010	1	7	0
NqA	2014	1	2	0
msgl	2014	1	1	0
PACRAT	2003	1	2	0
MLSeq	2017	1	0	0
miXGENE	2015	1	2	0
comTAR	2014	1	1	0
mirSTP	2017	1	0	0
miRprimer	2014	1	14	0
ESPSearch	2005	1	2	0
miRNA_Targets	2012	1	10	0
miRAFinder and GeneAFinder scripts	2014	1	0	0
miRseqViewer	2015	1	0	0
miTRATA	2016	1	4	0
START	2006	1	2	0
OfftargetFinder	2016	1	1	0
lncRInter	2017	1	0	0
The Porcine Translational Research Database	2017	1	0	0
NanoStringNorm	2012	1	39	0
findr	2017	1	0	0
Semirna	2012	1	2	0
miRandb	2017	1	0	0
OmicKriging	2014	1	13	0
MicroTrout	2016	1	1	0
dChip	2011	1	4	0
RiceChip	2010	1	0	0
MicroSyn	2011	1	11	0
Wormnet	2014	2	9	0

CoMoFinder	2015	1	1	0
dbPHCC	2016	1	0	0
GEISHA	2007	2	30	0
AraPath	2012	1	7	0
QuickMap	2009	1	18	0
DiseaseConnect	2014	1	16	0
PGnet	2009	1	4	0
miRNA-Analyzer	2016	1	0	0
biRte	2015	1	3	0
SNPLogic	2009	1	14	0
TF--miRNA	2016	1	0	0
SignaFish	2016	1	0	0
miRTarVis+	2017	1	0	0
GAMDB	2016	1	2	0
LncEnvironmentDB	2014	1	1	0
PD map	2014	1	26	0
PlanTE-MIR DB	2016	1	2	0
New support vector machine-based method for	2014	1	1	0
AmphiEST	2010	1	1	0
InCroMAP	2013	2	3	0
miRDis	2017	1	0	0
RNAComposer	2016	1	3	0
AmiRNA Designer	2016	1	1	0
NoiseMaker	2010	1	2	0
Pseudo-3D Clustering	2016	1	0	0
BCCTBbp	2015	1	3	0
webFOG	2010	1	0	0
FMIMS	2016	1	0	0
activeMiRNA	2013	1	3	0
LiverCancerMarkerRIF	2014	1	2	0
miRNA-ensemble	2017	1	0	0
Loregic	2015	1	1	0
miRPursuit	2017	1	0	0

rnaworkbench	2008	1	1	0
miRpower	2016	1	2	0
Carnation DB	2014	1	5	0
MULSEA	2016	1	0	0
UFFizi	2010	1	1	0
GeneFriends	2015	1	13	0
ParSel	2017	1	0	0
EDMRP	2014	1	1	0
MiRE	2008	1	0	0
miRNAfe	2015	1	3	0
deepSOM	2016	1	0	0
iJRF	2017	1	0	0
OligoMatcher	2006	1	0	0
mirnanalyze	2017	1	0	0
At_miRNA	2013	1	6	0
MirPlex	2013	1	3	0
mTD	2017	2	0	0
miRUPnet	2012	1	3	0
MTDB	2010	1	7	0
IsomiR Bank	2016	1	0	0
miRClassify	2014	1	17	0
miRTP	2010	1	2	0
SlideBase	2016	1	0	0
ncRNAppi	2009	1	0	0
plantMirP	2016	1	0	0
miRSeqNovel	2012	1	4	0
ImmunemiR	2017	2	0	0
JBCB	2010	1	7	0
MirCompare	2016	1	2	0
iBeetle-Base	2015	1	15	0
iSmaRT	2017	1	0	0
Mirin	2014	1	1	0
BosFinder	2014	1	0	0

DREAM	2015	1	3	0
SNPeffect and PupaSuite	2008	1	26	0
PlaMoM	2017	1	1	0
siRNAs with high specificity to the target	2008	1	2	0
EpimiRBase	2016	1	2	0
Radiogenomics	2015	1	8	0
application in consensus ranking of microRNA	2013	1	0	0
MethHC	2015	1	28	0
CHRONOS	2016	1	2	0
pirnaPre	2016	1	0	0
iScreen	2015	1	0	0
FREM	2016	1	0	0
TROD	2004	1	4	0
GED	2017	1	0	0
HCCNet	2010	1	7	0
